# Critical roles for EGFR and EGFR–HER2 clusters in EGF binding of SW620 human carcinoma cells

**DOI:** 10.1098/rsif.2022.0088

**Published:** 2022-05-25

**Authors:** Adam J. M. Wollman, Charlotte Fournier, Isabel Llorente-Garcia, Oliver Harriman, Alex L. Payne-Dwyer, Sviatlana Shashkova, Peng Zhou, Ta-Chun Liu, Djamila Ouaret, Jenny Wilding, Akihiro Kusumi, Walter Bodmer, Mark C. Leake

**Affiliations:** ^1^ Department of Physics, University of York, York, UK; ^2^ Department of Biology, University of York, York, UK; ^3^ Department of Physics, Clarendon Laboratory, University of Oxford, Oxford OX1 3PU, UK; ^4^ Science and Technology Group, Okinawa Institute of Science and Technology Graduate University (OIST), 1919 Tancha, Onna-son, Okinawa 904-0495, Japan; ^5^ Membrane Cooperativity Unit, OIST, 1919 Tancha, Onna-son, Okinawa 904-0495, Japan; ^6^ MRC Weatherall Institute of Molecular Medicine, University of Oxford, John Radcliffe Hospital, Oxford OX3 9DS, UK; ^7^ Biosciences Institute, Newcastle University, Newcastle upon Tyne NE2 4HH, UK

**Keywords:** single molecule, cancer, super-resolution, receptors, inhibitors

## Abstract

Epidermal growth factor (EGF) signalling regulates normal epithelial and other cell growth, with EGF receptor (EGFR) overexpression reported in many cancers. However, the role of EGFR clusters in cancer and their dependence on EGF binding is unclear. We present novel single-molecule total internal reflection fluorescence microscopy of (i) EGF and EGFR in living cancer cells, (ii) the action of anti-cancer drugs that separately target EGFR and human EGFR2 (HER2) on these cells and (iii) EGFR–HER2 interactions. We selected human epithelial SW620 carcinoma cells for their low level of native EGFR expression, for stable transfection with fluorescent protein labelled EGFR, and imaged these using single-molecule localization microscopy to quantify receptor architectures and dynamics upon EGF binding. Prior to EGF binding, we observe pre-formed EGFR clusters. Unexpectedly, clusters likely contain both EGFR and HER2, consistent with co-diffusion of EGFR and HER2 observed in a different model CHO-K1 cell line, whose stoichiometry increases following EGF binding. We observe a mean EGFR : EGF stoichiometry of approximately 4 : 1 for plasma membrane-colocalized EGFR–EGF that we can explain using novel time-dependent kinetics modelling, indicating preferential ligand binding to monomers. Our results may inform future cancer drug developments.

## 1. Introduction

Epidermal growth factor receptor (EGFR) is a cell surface receptor essential for cell growth and differentiation, with its disregulation implicated in several carcinomas [1], hence a target for numerous cancer drugs. Human EGFR or ERBB1 (ErB1 or HER1) is a protein of the receptor tyrosine kinase (RTK) family and the ERBB subfamily with three other ERBB members, ERBB2 (ErbB2 or HER2), ERBB3 (ErbB3 or HER3) and ERBB4 (ErbB4 or HER4), expressed in the plasma membranes of mainly epithelial cells [2]. EGFR has an extracellular region with subdomains I–IV, of which I and III participate in ligand binding [3]. The extracellular region is connected to a cytoplasmic domain containing a tyrosine kinase.

There are 11 ligands that can bind to ERBB proteins, including epidermal growth factor (EGF) which binds to EGFR [4]. Ligand binding induces receptor dimerization/clustering, resulting in activation following tyrosine residue autophosphorylation that initiates signalling reactions to stimulate cell growth, differentiation and proliferation. Structural evidence indicates that activation is preceded by EGF binding to EGFR monomers that induces a conformational change by removing interactions that autoinhibit EGFR dimerization [5]. Binding studies of full-length receptors suggest negative cooperativity, mediated through an intracellular juxta-membrane domain [6], as do radioligand-binding and phosphorylation assays [7,8].

An early single-molecule fluorescence imaging study using model human epidermoid cell line A431 published in 2000 reported binding of single EGF to a pre-formed EGFR dimer, followed by a second molecule to form a 2 : 2 complex [9]; however, later findings from *Xenopus* oocytes suggested in that system that the majority of EGFR was present as a monomer [10]. Other studies have instead reported observations of pre-formed EGFR oligomers using a range of methods comprising antibody-labelled EGF [11], Förster resonance energy transfer [12], autocorrelation analysis [13], bimolecular fluorescence complementation [14], pixel brightness analysis [15] and single-molecule live cell light microscopy [16,17]. The clustering and oligomeric states of EGFR are also complex since they may involve cooperativity not only between EGFR but also other ERBB proteins [14]. EGFR's clustering state before and after EGF binding under physiological conditions has remained contentious due to limitations in simultaneous data on stoichiometries of interacting receptors and ligands, to a dependence of EGF expression on EGFR clustering, to the common simultaneous presence of fluorescently labelled EGFR and dark EGFR, and to the existence of species-specific cell line differences.

Other ERBB receptors such as HER2 have been detected in monomeric, dimeric and higher-order clusters in human breast cancer cells [18], and in clusters of 2–4 HER2 molecules in fixed breast cancer cell lines determined using super-resolution fluorescence microscopy [19]. Furthermore, several light microscopy studies have suggested interactions of EGFR with other ERBB receptors in human cancer cells. For example, EGFR and HER2 co-express in human bladder cancer and colorectal cancer cell lines [20,21] and SKBR3 human breast cancer cells. In SKBR3, EGFR and HER2 expression levels can jointly increase in large membrane protrusions [22], hinting at the possibility of EGFR–HER2 heterodimers. EGFR-HER2 interactions inside lipid rafts in SKBR3 cells have also been proposed [23]. The presence of pre-formed homo- and heterodimers of different ERBB family members, including EGFR and HER2, has also been inferred from lysate analysis of transfected CHO cells [14]. Interactions of EGFR with the hepatocyte growth factor receptor HGFR (also known as MET) have also been inferred from single-molecule imaging where increased colocalization and decreased diffusion was observed in live HeLa and BT-20 cells after EGF stimulation [24].

**Figure 1.**
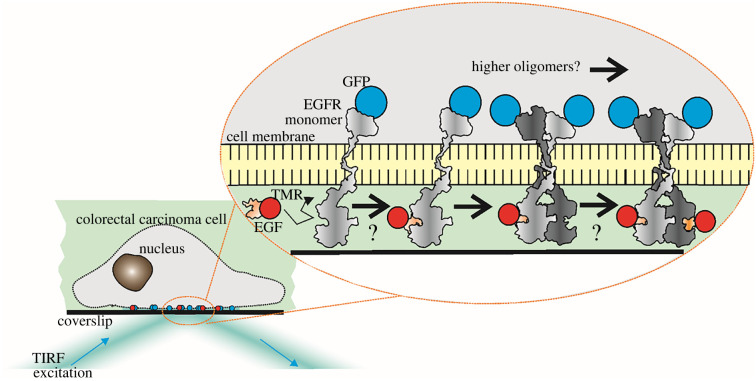
Visualizing EGF–EGFR in SW620 cells. Current models to explain EGFR activation encompass different binding rates of EGF to EGFR monomers and dimers, and binding cooperativity between EGF and EGFR. However, questions remain as to the role of EGFR clusters in cancer cells and their dependence on EGF binding. Here we used TIRF microscopy of GFP-labelled EGFR (blue) and TMR-labelled EGF (red) to enable SMLM to address these questions.

Here, we used two-colour single-molecule total internal reflection fluorescence (TIRF) microscopy for super-resolved single-molecule localization microscopy (SMLM) on live human colon carcinoma cells stably expressing EGFR–GFP in the presence of tetramethylrhodamine (TMR) conjugated to EGF (figure 1). Supported by predictions from Monte Carlo simulations, we find that prior to EGF binding, EGFR forms clusters with a modal stoichiometry of six molecules but extending to tens of molecules, adding to an emerging consensus that pre-formed EGFR clusters exist prior to EGF activation. Following EGF binding, we see clusters with a threefold higher stoichiometry. We find that EGF-bound EGFR clusters have a relative stoichiometry ratio for EGFR : EGF of approximately 4 : 1, which we interpret using a new time-dependent kinetics model that shows preferential ligand binding to receptor monomers with no binding to dimers. We present the first single-molecule light microscopy observations of the effect on live human cancer cells of anti-cancer immunotherapy drugs cetuximab [25] and trastuzumab [26] which specifically and separately inhibit EGFR activation by targeting either EGFR or HER2, respectively. We find that both promote an increase in EGFR cluster stoichiometry and a decrease in diffusion coefficient after the addition of EGF. Compared to untreated cells, treatment with either drug in addition to EGF results in increased numbers of EGFR molecules in a cluster and in a higher diffusion coefficient for EGF-bound EGFR clusters, which may reflect cluster compaction. Additionally, we present novel dual-colour single-molecule TIRF imaging of EGFR–HER2 interactions from live CHO-K1 cells that contain both fluorescently labelled EGFR and HER2. These data show that EGFR and HER2 in a model cell line interact transiently before EGF binding with a dwell time of several hundred milliseconds. Taken together, these observations show that EGFR clusters comprise a mixture of EGFR and HER2, to be compared with indirect findings of heterodimer formation in SKBR3 breast cancer cells from correlative fluorescence microscopy and liquid phase electron microscopy [22]. Our results provide new insights into architectures, dynamics and interactions of EGFR molecules overexpressed in carcinoma cells. Instead of a simplified picture for EGFR function in terms of monomer and dimer states, they indicate higher levels of complexity which hitherto has not been addressed explicitly. Given the nature of the EGFR pathway as an anti-cancer drug target, our results may inform the development of new therapeutic strategies to treat cancer.

## 2. Results

### 2.1. Single-molecule microscopy reveals epidermal growth factor receptor clusters before epidermal growth factor binds in SW620 cells

To visualize EGFR molecules in live cancer cells, we generated a human colon carcinoma cell line stably expressing EGFR–GFP. Immortalized cell line SW620, deriving from a human lymph node metastasis from an adenocarcinoma of the colon [27], was selected from a colon carcinoma library [28] for its very low EGFR expression (electronic supplementary material, figures S1 and S2) consistent with previous recent findings [29–32] and low expression of the most common EGFR ligands, including TGFA. TGFA has been reported to be expressed in SW620 cells from one study published in 1987 [33]; however, our recent high-precision microarray measurements indicate only very low levels (electronic supplementary material details our SW620 microarray results for all EGFR ligands). EGFR–GFP kinase activity in SW620 cells was confirmed by observing increased phosphorylation of EGFR downstream targets, ERK1/2, in response to EGF (electronic supplementary material, figure S2b).

We optimized a home-built TIRF microscope (electronic supplementary material, figure S4) for single-molecule detection, confirmed using an *in vitro* surface assay [34] in which GFP was antibody-conjugated to a glass coverslip (electronic supplementary material, figure S5). After approximately 1 s of laser illumination, bright spots (fluorescent foci) on our image sequences exhibited step-wise photobleaching (electronic supplementary material, figure S5) indicating the presence of single GFP molecules. Single fluorophore brightness values were quantified by analysing distributions of fluorescent foci intensity values (electronic supplementary material, figure S5c).

**Figure 2.**
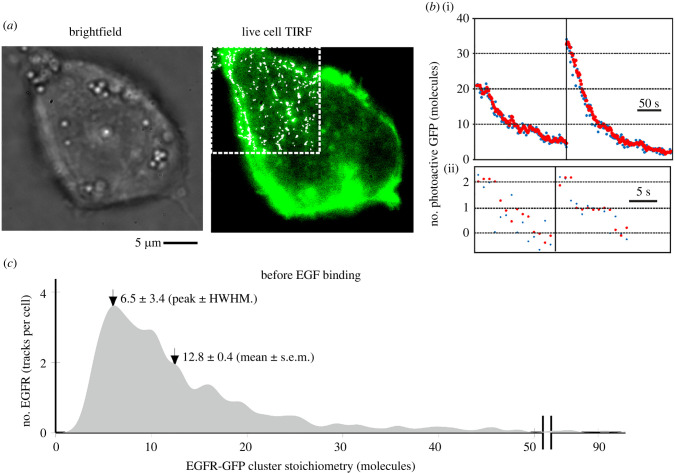
EGFR cluster stoichiometry in SW620 cells before EGF binding. (*a*) Transfected SW620 cell showing GFP (green) and overlaid tracking (white) on top left corner. (*b*) Photobleaching intensity traces from tracked EGFR–GFP clusters with stoichiometries of several tens of molecules (i), down to two molecules (ii), raw data (blue) and Chung-Kennedy filtered (red) [35,36] that preserves distinct edges such as those due to GFP photobleaching. (*c*) Distribution of EGFR cluster stoichiometry rendered as a kernel density estimation [37] before EGF binding showing peak at approximately six molecules and a mean of 12.8 molecules, with *N* = 19 cells, and 1250 cluster tracks in total (66 tracks per cell), corresponding to approximately 850 tracked EGFR per cell on average.

We applied our optimized TIRF microscopy to transfected SW620 cells in serum-free medium without the addition of EGF. We observed fluorescent foci at a surface density of 0.1–0.4 per µm^2^ in the basal plasma membrane in contact with the glass coverslip (figure 2*a*; electronic supplementary material, figure S6) with a mean of 66 ± 28 (s.d.) foci per cell. In most cells, foci could be detected across the full extent of the basal membrane and exhibited a smooth surface topography consistent with earlier scanning electron microscopy performed on SW620 cells [38]. We tracked foci over several seconds to approximately 40 nm spatial precision using home-written tracking software [39–45] (electronic supplementary material, movie S1).

Foci image widths were on average within 10% of those observed for single GFP *in vitro* (approx. 250 nm half width at half maximum). However, their brightness was greater than that expected for monomeric GFP, with fluorescence-intensity traces exhibiting multiple stochastic photobleaching steps (figure 2*b*) indicative of several molecules within each EGFR cluster. We could determine the stoichiometry of these foci by dividing their initial brightness by that of a single GFP [34]. The mean brightness of a single GFP was measured *in vivo* by quantifying the foci brightness towards the end of a photobleach trace, when only one photoactive molecule remained. The *in vivo* single-GFP brightness obtained in this way was within 15% of that measured *in vitro*, confirming accurate single-molecule detection *in vivo* (electronic supplementary material, figure S5c).

By integrating total fluorescence GFP intensity in each cell and correcting for native autofluorescence, we estimate the total copy number is approximately 200 000 EGFR–GFP molecules per cell. Tracked foci brightness values indicated that they comprise clusters of EGFR with a broad stoichiometry distribution, across different cells and within the same cell, with a range 2–90 EGFR molecules per cluster, with a peak value of approximately 6 and a mean of 12.8 ± 0.4 molecules (±s.e.m.) (figure 2*c*). We did not detect any monomeric EGFR–GFP before adding EGF from greater than 1000 tracks in 19 different cells (electronic supplementary material, table S1), despite our microscope having single-GFP sensitivity *in vivo* and *in vitro* under the same imaging conditions (electronic supplementary material, figure S5). We considered whether the absence of detected monomers and broad range of stoichiometry could be due to the random optical overlap of lower stoichiometry EGFR clusters in our diffraction-limited images. We modelled this effect by convolving a Poisson distribution calculated from the overlap probability [46] with the brightness distribution of a cluster in a range of different stoichiometry states (similar to earlier studies [17,47]). The simulated EGFR cluster stoichiometry distributions due to optical overlap for one–four molecules per cluster had a poor resemblance to the experimental stoichiometry distribution (electronic supplementary material, figure S7). However, simulating a cluster stoichiometry randomly sampled from a second Poisson distribution with peak value equal to four molecules per cluster, but extending to tens of molecules per cluster, resulted in reasonable predictions which could account for approximately 90% of the observed variance (*R*^2^ = 0.88) in the experimental stoichiometry distribution (electronic supplementary material, figure S7). This suggests that many EGFR foci are formed from clusters with a broad stoichiometry distribution. Rather than EGFR being a fixed, covalently bound tetramer, these results suggest a more loosely bound assembly of EGFR, comprising monomers and dimers that condense into clusters before EGF is added.

### 2.2. Epidermal growth factor causes clusters to increase their epidermal growth factor receptor content

To determine the effect of EGF binding on EGFR cluster stoichiometry and spatio-temporal dynamics, we performed TIRF following addition of EGF to the cell culture. We kept live SW620–EGFR–GFP cells in serum-free media for 24 h prior to imaging to minimize binding of serum-based EGFR ligands and then washed immediately prior to EGF addition. We then added EGF conjugated 1 : 1 with fluorescent tetramethylrhodamine (EGF–-TMR) at a final concentration of 100 ng ml^−1^ (15.6 nM), higher than the *K_D_* for EGF to EGFR of 300 pM–2 nM [48], and visualized cells using TIRF to allow simultaneous observation of EGFR and EGF in our green- and red-colour channels, respectively. Excess EGF–TMR was retained in the sample during imaging enabling observation over incubation times of 3–60 min.

**Figure 3.**
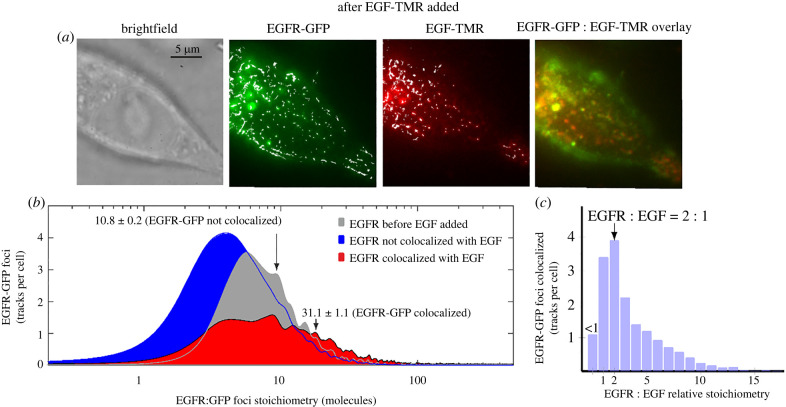
EGF increases EGFR cluster stoichiometry in SW620 cells. (*a*) Brightfield and TIRF of SW620–EGFR–GFP cells after adding EGF–TMR (10 min time point). GFP (green), TMR (red) foci and overlay images are shown with yellow indicating colocalization (putative binding between EGFR clusters and EGF within our 40 nm spatial precision). Overlaid tracks are shown (white). (*b*) Stoichiometry distributions of EGF-bound EGFR clusters (red) and EGFR not bound to EGF (blue) across all times. Mean and s.e.m. for each distribution indicated (arrows). (*c*) Distribution of relative EGFR : EGF stoichiometry for EGF-bound clusters. *N* = 117 cells.

Colocalization of EGFR and EGF foci was determined using the numerical overlap integral between tracked green/red foci, establishing a metric for putative binding of EGF to EGFR clusters to within our spatial precision of 40 nm. After EGF incubation for a few minutes, binding between green/red foci was detected (figure 3*a*; electronic supplementary material, movie S2 and figure S8). We observed a mean of approximately 57 EGFR tracks per cell across all incubation times from 117 cells and a total of 4700 tracks across all cells (electronic supplementary material, table S1). We estimated 40 ± 18% of EGFR clusters were bound to EGF over 3–60 min incubation, corresponding to 64% of all tracked EGFR clusters (electronic supplementary material, figure S9).

The EGFR stoichiometry for clusters not bound to EGF was similar to the value (approx. 13 molecules) measured before adding EGF (electronic supplementary material, table S1 and figure S9c; figure 3*b*). EGF-bound EGFR clusters had a higher mean stoichiometry of approximately 31 EGFR molecules compared to 11 EGFR molecules for clusters not bound to EGF, as shown in the electronic supplementary material, table S1, and the stoichiometry distributions in figure 3*b* (Student's *t-*test *p* < 0.0001), with non-parametric testing also indicating that the two distributions were statistically different (*p* < 0.0001). Binning the stoichiometry as a function of incubation time (electronic supplementary material, figure S9c), the mean stoichiometry of EGFR clusters not bound to EGF remained roughly constant at 8–14 during incubation with EGF over 60 min, whereas that of EGF-bound EGFR clusters increased to 20–50 molecules per cluster.

### 2.3. Epidermal growth factor-bound clusters contain four epidermal growth factor receptor molecules per epidermal growth factor

To determine the relative stoichiometry between EGFR clusters and EGF when EGF was bound, we measured red foci stoichiometry simultaneously to colocalized green foci. EGF stoichiometry was determined using the same photobleaching protocol to that of GFP-labelled EGFR. Fluorescence-intensity traces for EGF–TMR on the red channel exhibited step-wise photobleaching when multiple EGF–TMR molecules where present, and EGF foci stoichiometry was obtained by dividing the initial intensity in the traces by that of a single TMR molecule. As for GFP, the latter (approx. 2400 counts on our detector) was obtained *in vivo* from the final brightness in the photobleach, averaging over multiple traces, and agreed with *in vitro* measurements (electronic supplementary material, figure S5c). Our analysis revealed a modal relative stoichiometry for EGFR : EGF of 1.9 ± 0.8 (±half width half maximum; figure 3*c*) with mean 4.2 ± 0.1; EGFR clusters bound to EGF contain approximately four EGFR molecules for every EGF.

To interpret these observations, we developed a new multi-state time-dependent kinetics model that accounts for EGF–EGFR binding, receptor dimerization and receptor internalization and recycling (electronic supplementary material, figure S10a). The model predicts the fractional saturation on the surface, $Y^{\mathrm{surface}}$, which is the surface ratio EGF : EGFR (excluding internalized molecules). The model shows that on adding EGF, initial concentrations of unligated EGFR monomers ([R]) and dimers ([RR]) decrease while concentrations of ligated monomers ([RL]) and dimers (singly ligated [RRL] and doubly ligated [RRL2]) increase over the first 5 min (electronic supplementary material, figure S10a). Endocytosis leads to the accumulation of internalized ligated monomers ([RL^inside^]) and dimers (singly ligated [RRL^inside^] and doubly ligated dimers [RRL2^inside^]) (dashed lines, electronic supplementary material, figure S10a) with EGFR recycling back to the plasma membrane contributing to equilibration of all concentrations after approximately 30 min (electronic supplementary material, figure S10a). $Y^{\mathrm{surface}}$ is shown in the inset in electronic supplementary material, figure S10a. Its inverse at equilibrium predicts an EGFR : EGF ratio of approximately 1.5, lower than our observed mean value of approximately 4. However, if we assume that ligand can bind only to receptor monomers (and not to dimers), our model predicts $Y^{\mathrm{surface}}$ of 0.24, which corresponds closely to the experimental mean EGFR : EGF ratio of approximately 4 (electronic supplementary material, figure S10c,d).

### 2.4. Epidermal growth factor receptor clustering increases on adding cetuximab or trastuzumab

It is known that EGF binds to monomeric EGFR resulting in EGFR dimerization prior to activation [5–8]; however, it is less clear what role EGFR activation plays in EGFR clustering. To investigate the effect of EGFR pathway inhibition on EGFR clustering, we imaged the transfected SW620 cells in the presence of EGFR pathway inhibitors cetuximab or trastuzumab, two commonly used anti-cancer drugs, which separately target EGFR and HER2, respectively. These are, to our knowledge, the first single-molecule observations of the effect of EGFR- and HER2-targeting anti-cancer drugs on living human cancer cells. Cetuximab targets EGFR and is a monoclonal antibody anti-cancer drug commonly used against neck and colon cancers in advanced disease stages to inhibit cell division and growth [25]. Binding of cetuximab to domain III of the soluble extracellular segment of EGFR is believed to result in partial blockage of the EGF-binding region, hindering the adoption of an extended conformation required for EGFR dimerization. Trastuzumab is a monoclonal antibody anti-cancer drug commonly used to treat breast cancer [26] that results in similar downstream effects of EGFR pathway inhibition of impairing cell division and growth. However, trastuzumab does not bind directly to EGFR but to domain IV of the extracellular segment of HER2 [49]. Trastuzumab binding does not affect HER2 self-association [50] but influences the stability of HER2-mediated dimers with EGFR [51].

**Figure 4.**
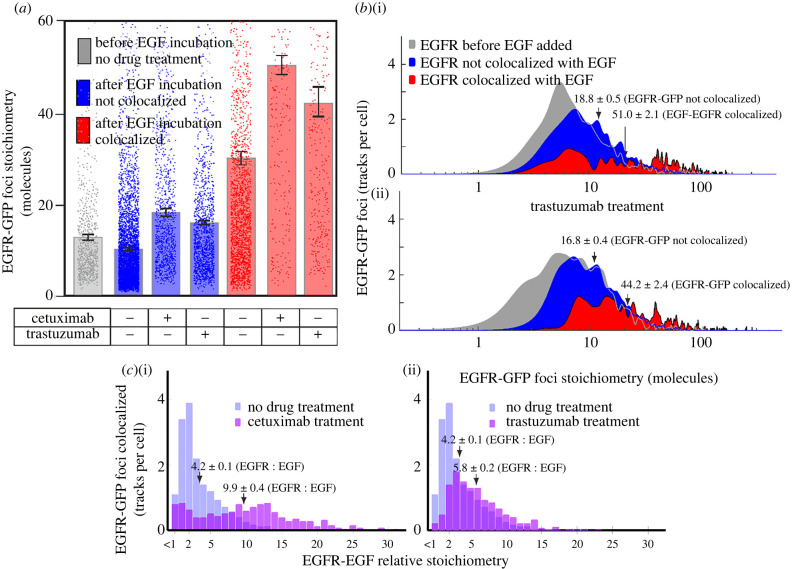
Cetuximab and trastuzumab increase EGFR cluster stoichiometry. (*a*) Mean EGFR cluster stoichiometry before and after EGF incubation and its dependence on EGF binding, in the presence (+) or absence (−) of each drug treatment. Error bars are s.e.m, *N* = 10–117 cells per dataset (see electronic supplementary material, table S1). (*b*) Distributions of EGFR cluster stoichiometry for cells treated with cetuximab (i) or trastuzumab (ii). Distributions shown are pre-EGF addition (grey) and post-EGF addition for EGF-bound EGFR clusters (red) and EGFR not bound to EGF (blue). Data collated across 60 min EGF incubation. Mean and s.e.m. values are indicated by arrows. (*c*) Distributions of EGFR : EGF relative stoichiometries of EGF-bound EGFR clusters for drug-treated cells (purple) contrasted against no drug treatment (light blue). *N* = 10–117 cells per dataset.

Before adding EGF, we found that treatment with cetuximab or trastuzumab at cytostatic concentrations caused statistically significant differences between the stoichiometry distributions for EGFR–GFP stoichiometry (Student's *t-*test, *p* < 0.0001, Brunner–Munzel, *p* = 0.01, *p* = 0.08, respectively) but with no significant effect on the number of detected EGFR–GFP tracks per cell (electronic supplementary material, table S1). EGF incubation together with drug treatment resulted in increased EGFR cluster stoichiometries for both EGF-bound and EGFR-unbound clusters, for both drugs, compared to stoichiometries after EGF incubation with no drug treatment (figure 4*a*,*b*; electronic supplementary material, table S1). The mean stoichiometry of EGF-bound EGFR clusters in drug-treated cells increased significantly to 51 ± 2 and 44 ± 2 for cetuximab and trastuzumab, respectively, with maxima of several hundred molecules (figure 4*a*,*b*; electronic supplementary material, table S1). There were approximately 20% fewer EGF-bound EGFR tracks for cetuximab- or trastuzumab-treated cells compared to untreated cells (electronic supplementary material, figure S12). We also observed a shift to higher EGFR : EGF relative stoichiometry for cetuximab and trastuzumab treatments beyond the approximately 2 : 1 modal ratio observed for untreated cells (figure 4*c*), consistent with competitive inhibition of EGF binding. Taken together, these results support the hypothesis that EGF binding increases the level of EGFR clustering.

### 2.5. Epidermal growth factor triggers larger epidermal growth factor receptor heterocluster formation

**Figure 5.**
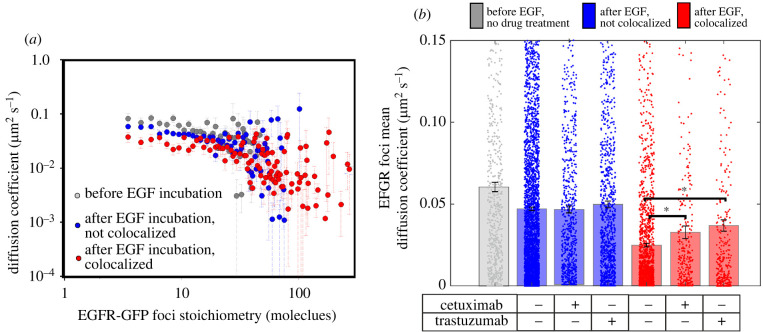
EGFR cluster diffusion depends on stoichiometry and EGF binding. (*a*) Log–log plot for apparent diffusion coefficient, *D*, as a function of EGFR cluster stoichiometry. (*b*) *D* upon drug treatment for the same datasets of figure 4. Significant differences using Student's *t*-test (*p* < 0.05) for + cetuximab and + trastuzumab (*p* = 0.01 and less than 0.0001) are indicated with asterisk, with corresponding Brunner–Munzel tests on the full distributions indicating *p*-values of 0.0001 and 0.001, respectively; s.e.m., error bars.

Tracking of EGFR clusters indicated Brownian diffusion up to time intervals of approximately 100 ms (electronic supplementary material, figure S13). Using the initial gradient of the mean square displacement with respect to time interval for each track, we determined the apparent diffusion coefficient *D* and correlated this against EGFR cluster stoichiometry. Plotting *D* against stoichiometry for all tracked clusters shows a trend towards lower diffusion with higher stoichiometry (figure 5*a*; electronic supplementary material, figures S13 and S14). One explanation for these observations can be made using the principles of the Stokes–Einstein relation, which states that *D* = *k_B_T*/*γ*, where *k_B_* is Boltzmann's constant, *T* is the absolute temperature and *γ* is the frictional drag of a tracked EGFR cluster in the membrane. The frictional drag is dependent on the local viscosity and the size and shape of the diffusing object. Larger clusters (i.e. those with a higher effective diameter) have a higher frictional drag in the membrane so a trend towards lower cluster diffusion with higher number of EGFR molecules per cluster is not unreasonable for an accretion model of cluster growth. In the absence of any drugs, *D* for EGF-bound clusters was lower than that for EGF-unbound clusters (red data to blue, figure 5*b*), that would be consistent with an increase in effective cluster diameter as clusters accumulate more EGFR upon EGF binding triggering increased dimerization. However, for clusters that have grown much larger than the approximately 3–5 nm width of the two-dimensional cell membrane, there is an expectation that the effective drag coefficient has a less-sensitive logarithmic scaling with effective diameter as opposed to being inversely proportional to the effective diameter of an object diffusing in a purely three-dimensional environment [52], so there may be additional effects to consider (see Discussion).

We found that the addition of cetuximab or trastuzumab made relatively little difference to *D* for EGF-unbound EGFR clusters (blue data, figure 5*b*), suggesting that these drug treatments are unlikely to have a significant effect on the plasma membrane viscosity in the vicinity of EGFR clusters, or on interactions of EGFR with the cytoskeleton that could influence cluster diffusion. However, we also found that both cetuximab and trastuzumab increased *D* for EGF-bound EGFR clusters (red data, figure 5*b*) in the direction of higher values associated with EGFR clusters not bound to EGF in the untreated datasets (grey bar, figure 5*b*). One hypothesis for these findings is that there are non-EGFR components present in clusters that influence *D*. HER2 is a candidate here, since trastuzumab binds not to EGFR but specifically to HER2; since the frictional drag of an EGFR cluster includes not only visible GFP-labelled EGFR but also any unlabelled components that contribute to frictional drag, one explanation is that trastuzumab reduces the EGFR cluster diameter by perturbing the association between EGFR and unlabelled HER2 if present in a cluster, following EGF binding to EGFR. In support of this explanation, HER2 is known to affect the stability of HER2-mediated dimers with EGFR [51] while not affecting the binding of HER2 with other HER2 molecules [50]. An important conclusion to this hypothesis is that it is likely that, prior to drug treatment, there must clusters present that comprise both EGFR and HER2, i.e. heteroclusters.

A number of previous findings have inferred indirectly that EGFR may form heterocomplexes with other RTKs [14,20,22–24], and recent evidence shows that HER2 inhibitor lapatinib induces HER2/HER3 heterocomplex formation in breast cancer cells [53].

We have no available viable cell line derived from the SW620 cell line currently that has both HER2 and EGFR fluorescently labelled; however, we were able to construct a dual-label cell line using model CHO-K1 cells that have similar low endogenous EGFR expression levels. We constructed this cell line to contain GFP-labelled HER2 and EGFR labelled with HaloTag650 (HaloTag STELLA Fluor 650) ligand. Using similar TIRF and SMLM, we found that HER2 and EGFR exhibit mobile and immobile foci, with transient colocalization and co-diffusion (electronic supplementary material, figure S15a) over a mean dwell time of 335 ± 100 ms (electronic supplementary material, figure S15b, movies S3 and S4). Although there are several biological differences between SW620 and CHO-K1 cells, taken together, our results add support to the hypothesis that EGF induces the formation of larger EGFR heteroclusters that involve a HER2 component (electronic supplementary material, figure S15c). Heteroclusters may also include HER3 or HER4. We tested this indirectly by treating cells with the inhibitor pertuzumab, a monoclonal antibody anti-cancer drug similar to trastuzumab albeit with complementary function against HER2/HER3 heteroassociation [54]. We found that pertuzumab treatment also resulted in perturbations to EGFR clustering (electronic supplementary material, figure S16), perhaps suggesting HER3 contribution to heteroclusters.

## 3. Discussion

Here, we investigated the role of EGFR clusters in cancer and their dependence on EGF binding. Two important improvements over earlier reports are (i) our SW620 observations relate to a human carcinoma line, enabling insights to the EGF pathway in cancer directly and (ii) we have spatial information concerning EGFR and EGF localization simultaneously from labelled protein and ligand. In prior microscopy in which labelled EGF is not imaged simultaneously to labelled EGFR, inference is more limited.

We used single-molecule TIRF and SMLM on transfected SW620 cells which do not natively express EGFR. By using GFP on EGFR with TMR on EGF, we have measured the stoichiometry and diffusion of single EGFR clusters, and how these depend on putative EGF binding within our 40 nm spatial precision. We find that before EGF binds to EGFR, EGFR comprises clustered assemblies, the most prevalent of which contains six EGFR molecules, but with the cluster stoichiometry extending to several tens of molecules. We find that binding of EGF to EGFR results in higher cluster stoichiometry. The observation that EGFR may exist as pre-formed clusters prior to EGF activation has been suggested previously by several studies [11–15,17] with our findings here adding to this growing consensus.

We developed a new time-dependent kinetics model using realistic parameters derived from previous studies. Unlike previous models, it predicts the time evolution of all concentrations and accounts for recycling and endocytosis. The model enables the interpretation of imaging data revealing insights that could not be achieved with time-independent schemes based solely on affinities and equilibrium constants. It also factors in the temperature dependence of EGF binding in living cells, showing the contrast between EGF : EGFR predictions at 37°C and 4°C ($Y^{\mathrm{surface}}∼0.24$ at 37°C versus $Y^{\mathrm{surface}}∼0.96$ at 4°C). These differences arise from the fact that receptor–EGF binding and dimerization equilibrium constants can strongly depend on temperature (they may vary by as much as a factor of 10–100 between approximately 0°C and 37°C [55]), as well as from the fact that receptor internalization is highly temperature dependent [56]. It is worth noting that our model has validity for any receptor–ligand system for which reaction rates have been measured. It predicts a mean EGFR : EGF ratio of 4 : 1 within a cluster which agrees with our experimental measurements. The model predications are not explicitly dependent on the presence of heterodimers. Limited experimental data from heterodimeric components for EGF activation rates and internalization processes preclude a full theoretical description within the current model framework, though it is not inconceivable that EGF-binding processes might be reaction-limited as opposed to diffusion-limited due to the relatively high rates of diffusion of the small EGF ligand comparable to EGFR clusters. In such a scenario, an EGFR molecule that is paired with a non-EGFR ERBB superfamily partner (for example, HER2, -3 or -4) might have comparable reaction kinetics to pure monomeric EGFR, though testing this is beyond the scope of our present study. To our knowledge, this is the first report of a truly time-dependent kinetics model applied to single-molecule precise live cell data; therefore, its accurate prediction adds significant support to the model's key premise that there is preferential EGF binding to EGFR monomers with no binding to dimers.

Our model adds to existing evidence of ligand binding to EGFR monomers. Small angle X-ray scattering and isothermal titration calorimetry to EGFR's isolated extracellular domain (sEGFR) suggest EGF binds to sEGFR monomers, receptor dimerization involving the association of two monomeric EGF–sEGFR [57]. Multi-angle laser light scattering suggests sEGFR is monomeric in solution but dimeric after EGF ligation [58]. Fluorescence anisotropy indicates 1 : 1 binding of EGF : sEGFR, analytical ultracentrifugation suggesting two (EGF–sEGFR) complexes [59]. Structural evidence indicates activation is preceded by ligand binding to receptor monomers [60–62].

We also performed TIRF with SMLM to investigate anti-cancer drugs cetuximab [25] and trastuzumab [26], to our knowledge for the first time on living human cancer cells, although correlative fluorescence microscopy and liquid phase electron microscopy have been used previously to investigate the drug lapatinib that reversibly inhibits both EGFR and HER2 [22]. We discovered that the diffusion of EGF-bound EGFR clusters increased upon treating cells with either drug. Since cetuximab and trastuzumab separately target EGFR and HER2, respectively [25,49], a reasonable conclusion is that clusters likely contain a mixture of both proteins.

One implication of this observation is that these drugs reduce the effective frictional drag experienced by clusters which could imply a compaction effect, i.e. that the EGFR packing density within EGF-bound clusters is higher for drug-treated compared to untreated cells. It is known that EGFR adopts a spatially more extended conformation for dimerization to occur [63]—when EGF binds to EGFR, the activated EGFR dimers become more compact than non-EGF-bound EGFR dimers in the two-dimensional plane of the plasma membrane, but also become marginally taller perpendicular to this plane. Therefore, if a cluster contains a mixture of both EGF-bound and EGFR-unbound subunits then the addition of a dimerization inhibitor might conceivably result in EGF-unbound EGFR subunits adopting the more compact conformation not associated with dimerization in the two-dimensional plane of the plasma membrane, so increasing the overall packing density of EGFR in that cluster. However, equivalent details are not currently known for trastuzumab. An alternative explanation is that there are changes to the membrane or cytoskeletal microenvironment in the vicinity of EGFR clusters that are dependent not only on the presence of the drugs used here but also on whether EGF is bound to EGFR. One further consideration concerns putative hop diffusion that was reported as a model to explain the apparent increases in translational diffusion for E-cadherin oligomers in the plasma membrane [64]. In this model, the plasma membrane is compartmentalized by the actin-based cytoskeleton into corral zones of a few hundred nanometre effective diameter that E-cadherin can hop between such that the hopping rate decreases dramatically with an increase of E-cadherin's oligomeric state. However, the spatial and temporal resolution limitations in our current work preclude us from probing this level of ultrastructural detail at this time.

Although we do not have a cell line in SW620 that co-expresses both fluorescently labelled EGFR and HER2, we were able to make a viable dual-label strain in model CHO-K1 cells, which indicated that EGFR and HER2 foci co-diffuse over periods of several hundred milliseconds prior to incubation with EGF. With the caveat that there are biological differences between SW620 and CHO-K1, if the molecular behaviours of EGFR and HER2 molecular interactions *per se* are fundamentally identical irrespective of the cell line, these data suggest that EGFR clusters may contain a mixture of EGFR and HER2 both before and after EGF binding. It should be noted that other reports suggest that HER2 and HER3 may engage in heterocomplex formation [53] so we cannot exclude the possibility that HER3 may also be present in mixed clusters of the SW620 line. Indeed, we tested this possibility indirectly by treating cells with pertuzumab, a monoclonal antibody anti-cancer drug which targets the HER2/HER3 interface. This treatment also induced stoichiometry changes to receptor clusters, suggesting a role for HER3 in heteroclusters; however, the full extent of HER3 and HER4 involvement in heteroclusters is beyond the scope of this present study.

Our findings show that EGFR is clustered before and after EGF binding, consistent with observations from AFM studies using EGF-coated tips which imaged human lung adenocarcinoma cells from the A549 cell line, known to have high EGFR expression [65]. These data suggested half the EGFR clusters had diameters of 20–70 nm pre-activation, with 35–105 nm post-activation, indicating cluster growth following EGF binding, to be compared with our findings. However, we find important differences with respect to some recent single-molecule studies. Although there were earlier suggestions of pre-formed oligomeric EGFR, including Needham *et al.* [17] and Zanetti-Domingues *et al.* [66], they and Huang *et al.* [10] observed monomeric EGFR, in particular Huang *et al.* assigning a proportion of 94%. We cannot directly exclude the possibility in our experiments that monomeric EGFR is at high density for which the mean separation is less than the optical resolution limit. However, the absence of not a single detected monomer from several thousand tracks, despite having single-GFP detection sensitivity, makes this unlikely. An alternative explanation is that we estimate the EGFR copy number to be approximately 200 000 molecules per cell, similar to endogenously expressing cancer cell lines [67] but more than double that estimated from Needham *et al.* and Huang *et al.*, which may conceivably result in shifting the equilibrium position for EGFR clustering towards higher stoichiometry. In support of this, the peak value of 6 EGFR cluster molecules we measure before EGF binds is higher than that observed by Needham *et al.* who observed 4. Such an increased on-rate could conceivably contribute to a depleted monomeric population, which has implications for carcinomas in which the expression level of EGFR is known to be high. The absence of monomeric EGFR before the addition of EGF may also suggest some spontaneous activation independent of ligand binding.

Reports on possible heterocomplex formation between EGFR and other ERBB proteins do not detail whether these associations are before or after EGF binding. Our observations show that transient associations between EGFR and HER2 may last a few hundred milliseconds, but that cluster size and number increase following EGF binding. Our findings suggest a role for trastuzumab in modulating regulatory balance through the availability of endogenous HER2 to associate with EGFR. Even when scarce, the presence of HER2 is known to selectively discourage internalization and degradation of activated EGFR and promote recycling to the plasma membrane via both chaperone proteins and EGF dissociation [68]. However, although HER2 is known to act as co-receptor, it has no known direct ligand and its physiological role in interacting with EGFR is still unclear. One possibility is that the diffusion of heterocomplexes may enable a spread of activation across cell surfaces. Also, the resistance of HER2-bearing complexes to downregulation might sustain signalling once established, i.e. a ‘latch’ response. Future work involving the development of a viable SW620 cell line that co-expresses labelled EGFR and HER2 may help these questions to be addressed, in particular to determine what ERBB component EGF specifically targets in clusters that contain heterodimers.

Future work will also be valuable to unravel how EGFR–HER2 heterocluster formation affects and is affected by the downstream signalling proteins, which themselves may cluster and alter their diffusion as has been observed in Ras signalling which interacts with EGFR [69]. Similar bidirectional effects occur with the cytoskeleton and through endocytosis. Constraining EGFR clustering and diffusion modulates phosphorylation [70], similarly inhibiting endocytosis increases EGFR autophosphorylation [71]. Unravelling the complex interplay between receptor clustering and diffusion with downstream signalling proteins, cytoskeletal interactions and endocytosis will remain a significant challenge going forward.

Our findings that heterocomplex cluster size increases post-EGF binding suggest new strategies for anti-cancer drug design. For example, new drugs to disrupt interface between HER2 and EGFR directly. Strategies that disrupt EGFR clusters before EGF binding may also inspire new drug designs. Similarly, single-molecule quantification would be valuable to probe different carcinomas, for example, those of the lung in which EGFR mutations are implicated in cancer [72] or in the design of cell surface logic gates for targeted therapies [73]. Finally, in enabling quantification of the actions of different drugs, there may be value in identifying chemotherapy ‘sweet-spots’ in carcinomas known to be treatable using combined drugs, such as in gastric cancer [74].

## 4. Material and methods

Full details for methods used for cell line preparation, gene expression quantification, microscopy and modelling are given in the electronic supplementary material.

### 4.1. Software access

All bespoke code in MATLAB is available at EGFRanalyser: https://sourceforge.net/projects/york-biophysics/.

## Data Availability

Analysed data are included in full in the main text and electronic supplementary material. All raw imaging data are available from the authors. The data are provided in the electronic supplementary material [75].

## References

[1] Roskoski R. 2014 The ErbB/HER family of protein-tyrosine kinases and cancer. Pharmacol. Res. **79**, 34-74. (10.1016/j.phrs.2013.11.002) 24269963

[2] Jorissen RN, Walker F, Pouliot N, Garrett TP, Ward CW, Burgess AW. 2003 Epidermal growth factor receptor: mechanisms of activation and signalling. Exp. Cell Res. **284**, 31-53. (10.1016/S0014-4827(02)00098-8) 12648464

[3] Lax I, Bellot F, Howk R, Ullrich A, Givol D, Schlessinger J. 1989 Functional analysis of the ligand binding site of EGF-receptor utilizing chimeric chicken/human receptor molecules. EMBO J. **8**, 421-427. (10.1002/j.1460-2075.1989.tb03393.x) 2785915 PMC400822

[4] Schneider MR, Wolf E. 2009 The epidermal growth factor receptor ligands at a glance. J. Cell Physiol. **218**, 460-466. (10.1002/jcp.21635) 19006176

[5] Ferguson KM, Berger MB, Mendrola JM, Cho HS, Leahy DJ, Lemmon MA. 2003 EGF activates its receptor by removing interactions that autoinhibit ectodomain dimerization. Mol. Cell **11**, 507-517. (10.1016/S1097-2765(03)00047-9) 12620237

[6] Macdonald-Obermann JL, Pike LJ. 2009 The intracellular juxtamembrane domain of the epidermal growth factor (EGF) receptor is responsible for the allosteric regulation of EGF binding. J. Biol. Chem. **284**, 13 570-13 576. (10.1074/jbc.M109.001487) PMC2679458 19336395

[7] Liu P, Cleveland TE, Bouyain S, Byrne PO, Longo PA, Leahy DJ. 2012 A single ligand is sufficient to activate EGFR dimers. Proc. Natl Acad. Sci. USA **109**, 10 861-10 866. (10.1073/pnas.1201114109) PMC3390854 22699492

[8] Macdonald JL, Pike LJ. 2008 Heterogeneity in EGF-binding affinities arises from negative cooperativity in an aggregating system. Proc. Natl Acad. Sci. USA **105**, 112-117. (10.1073/pnas.0707080105) 18165319 PMC2224169

[9] Sako Y, Minoghchi S, Yanagida T. 2000 Single-molecule imaging of EGFR signalling on the surface of living cells. Nat. Cell Biol. **2**, 168-172. (10.1038/35004044) 10707088

[10] Huang Y et al. 2016 Molecular basis for multimerization in the activation of the epidermal growth factor receptor. Elife **5**, e14107. (10.7554/eLife.14107) 27017828 PMC4902571

[11] Ichinose J, Murata M, Yanagida T, Sako Y. 2004 EGF signalling amplification induced by dynamic clustering of EGFR. Biochem. Biophys. Res. Commun. **324**, 1143-1149. (10.1016/j.bbrc.2004.09.173) 15485674

[12] Martin-Fernandez M, Clarke DT, Tobin MJ, Jones SV, Jones GR. 2002 Preformed oligomeric epidermal growth factor receptors undergo an ectodomain structure change during signaling. Biophys. J. **82**, 2415-2427. (10.1016/S0006-3495(02)75585-9) 11964230 PMC1302032

[13] Clayton AHA, Walker F, Orchard SG, Henderson C, Fuchs D, Rothacker J, Nice EC, Burgess AW. 2005 Ligand-induced dimer-tetramer transition during the activation of the cell surface epidermal growth factor receptor: a multidimensional microscopy analysis. J. Biol. Chem. **280**, 30 392-30 399. (10.1074/jbc.M504770200) 15994331

[14] Tao RHH, Maruyama IN. 2008 All EGF(ErbB) receptors have preformed homo- and heterodimeric structures in living cells. J. Cell Sci. **121**, 3207-3217. (10.1242/jcs.033399) 18782861

[15] Nagy P, Claus J, Jovin TM, Arndt-Jovin DJ. 2010 Distribution of resting and ligand-bound ErbB1 and ErbB2 receptor tyrosine kinases in living cells using number and brightness analysis. Proc. Natl Acad. Sci. USA **107**, 16 524-16 529. (10.1073/pnas.1002642107) 20813958 PMC2944731

[16] Kozer N, Barua D, Orchard S, Nice EC, Burgess AW, Hlavacek WS, Clayton AHA. 2013 Exploring higher-order EGFR oligomerisation and phosphorylation: a combined experimental and theoretical approach. Mol. Biosyst. **9**, 1849-1863. (10.1039/c3mb70073a) 23629589 PMC3698845

[17] Needham SR et al. 2016 EGFR oligomerization organizes kinase-active dimers into competent signalling platforms. Nat. Commun. **7**, 13307. (10.1038/ncomms13307) 27796308 PMC5095584

[18] Peckys DB, Korf U, De Jonge N. 2015 Local variations of HER2 dimerization in breast cancer cells discovered by correlative fluorescence and liquid electron microscopy. Sci. Adv. **1**, e1500165. (10.1126/sciadv.1500165) 26601217 PMC4646781

[19] Tobin SJ, Wakefield DL, Jones V, Liu X, Schmolze D, Jovanović-Talisman T. 2018 Single molecule localization microscopy coupled with touch preparation for the quantification of trastuzumab-bound HER2. Sci. Rep. **8**, 15154. (10.1038/s41598-018-33225-0) 30310083 PMC6181918

[20] Siddiqui MR, Railkar R, Sanford T, Crooks DR, Eckhaus MA, Haines D, Choyke PL, Kobayashi H, Agarwal PK. 2019 Targeting epidermal growth factor receptor (EGFR) and human epidermal growth factor receptor 2 (HER2) expressing bladder cancer using combination photoimmunotherapy (PIT). Sci. Rep. **9**, 2084. (10.1038/s41598-019-38575-x) 30765854 PMC6375935

[21] Ashraf SQ, Nicholls AM, Wilding JL, Ntouroupi TG, Mortensen NJ, Bodmer WF. 2012 Direct and immune mediated antibody targeting of ERBB receptors in a colorectal cancer cell-line panel. Proc. Natl Acad. Sci. USA **109**, 21 046-21 051. (10.1073/pnas.1218750110) PMC3529069 23213241

[22] Weinberg F, Peckys DB, de Jonge N. 2020 Egfr expression in her2-driven breast cancer cells. Int. J. Mol. Sci. **21**, 1-19. (10.3390/ijms21239008) PMC7729501 33260837

[23] Dahmke IN, Trampert P, Weinberg F, Mostajeran Z, Lautenschläger F, De Jonge N. 2020 Correlative fluorescence- and electron microscopy of whole breast cancer cells reveals different distribution of ErbB2 dependent on underlying actin. Front. Cell Dev. Biol. **8**, 521. (10.3389/fcell.2020.00521) 32714928 PMC7344305

[24] Harwardt MLIE et al. 2020 Single-molecule super-resolution microscopy reveals heteromeric complexes of MET and EGFR upon ligand activation. Int. J. Mol. Sci. **21**, 2803. (10.3390/ijms21082803) PMC7215329 32316583

[25] Kirkpatrick P, Graham J, Muhsin M. 2004 Fresh from the pipeline: cetuximab. Nat. Rev. Drug Discov. **3**, 549-550. (10.1038/nrd1445) 15272498

[26] Garnock-Jones KP, Keating GM, Scott LJ. 2010 Trastuzumab. Drugs **70**, 215-239. (10.2165/11203700-000000000-00000) 20108993

[27] Leibovitz A, Stinson JC, McCombs WB, McCoy CE, Mazur KC, Mabry ND. 1976 Classification of human colorectal adenocarcinoma cell lines. Cancer Res. **36**, 4562-4569. (10.1016/b978-0-12-043050-5.50047-0) 1000501

[28] Wilding JL, McGowan S, Liu Y, Bodmer WF. 2010 Replication error deficient and proficient colorectal cancer gene expression differences caused by 3′UTR polyT sequence deletions. Proc. Natl Acad. Sci. USA **107**, 21 058-21 063. (10.1073/pnas.1015604107) PMC3000307 21097699

[29] Frederiksen KS, Abrahamsen N, Cristiano RJ, Damstrup L, Poulsen HS. 2000 Gene delivery by an epidermal growth factor/DNA polyplex to small cell lung cancer cell lines expressing low levels of epidermal growth factor receptor. Cancer Gene Ther. **7**, 262-268. (10.1038/sj.cgt.7700098) 10770635

[30] Dahan L, Sadok A, Formento JL, Seitz JF, Kovacic H. 2009 Modulation of cellular redox state underlies antagonism between oxaliplatin and cetuximab in human colorectal cancer cell lines. Br. J. Pharmacol. **158**, 610-620. (10.1111/j.1476-5381.2009.00341.x) 19732064 PMC2757701

[31] Shih YH, Peng CL, Lee SY, Chiang PF, Yao CJ, Lin WJ, Luo TY, Shieh MJ. 2015 ^111^In-cetuximab as a diagnostic agent by accessible epidermal growth factor (EGF) receptor targeting in human metastatic colorectal carcinoma. Oncotarget **6**, 16 601-16 610. (10.18632/oncotarget.3968) PMC4599292 26062654

[32] Yang JL, Qu XJ, Russell PJ, Goldstein D. 2004 Regulation of epidermal growth factor receptor in human colon cancer cell lines by interferon a. Gut **53**, 123-129. (10.1136/gut.53.1.123) 14684586 PMC1773946

[33] Coffey RJ, Goustin AS, Soderquist AM, Shipley GD, Wolfshohl J, Carpenter G, Moses HL. 1987 Transforming growth factor *α* and *β* expression in human colon cancer lines: implications for an autocrine model. Cancer Res. **47**, 4590-4594. 2887281

[34] Leake MC, Chandler JH, Wadhams GH, Bai F, Berry RM, Armitage JP. 2006 Stoichiometry and turnover in single, functioning membrane protein complexes. Nature **443**, 355-358. (10.1038/nature05135) 16971952

[35] Leake MC, Wilson D, Bullard B, Simmons RM. 2003 The elasticity of single kettin molecules using a two-bead laser-tweezers assay. FEBS Lett. **535**, 55-60. (10.1016/S0014-5793(02)03857-7) 12560078

[36] Leake MC, Wilson D, Gautel M, Simmons RM. 2004 The elasticity of single titin molecules using a two-bead optical tweezers assay. Biophys. J. **87**, 1112-1135. (10.1529/biophysj.103.033571) 15298915 PMC1304451

[37] Leake MCC. 2014 Analytical tools for single-molecule fluorescence imaging in cellulo. Phys. Chem. Chem. Phys. **16**, 12 635-12 647. (10.1039/C4CP00219A) 24626744

[38] Palmieri V et al. 2015 Mechanical and structural comparison between primary tumor and lymph node metastasis cells in colorectal cancer. Soft Matter **11**, 5719-5726. (10.1039/C5SM01089F) 26083581

[39] Wollman AJM, Leake MC. 2015 Millisecond single-molecule localization microscopy combined with convolution analysis and automated image segmentation to determine protein concentrations in complexly structured, functional cells, one cell at a time. Faraday Discuss. **184**, 401-424. (10.1039/C5FD00077G) 26419209

[40] Miller H, Zhou Z, Wollman AJM, Leake MC. 2015 Superresolution imaging of single DNA molecules using stochastic photoblinking of minor groove and intercalating dyes. Methods **88**, 81-88. (10.1016/j.ymeth.2015.01.010) 25637032

[41] Wollman AJM, Shashkova S, Hedlund EG, Friemann R, Hohmann S, Leake MC. 2017 Transcription factor clusters regulate genes in eukaryotic cells. Elife **6**, e27451. (10.7554/eLife.27451) 28841133 PMC5602325

[42] Shashkova S, Wollman AJM, Leake MC, Hohmann S. 2017 The yeast Mig1 transcriptional repressor is dephosphorylated by glucose-dependent and independent mechanisms. FEMS Microbiol. Lett. **364**, fnx133. (10.1093/femsle/fnx133) 28854669

[43] Syeda AH, Wollman AJM, Hargreaves AL, Howard JAL, Brüning JG, Mcglynn P, Leake MC. 2019 Single-molecule live cell imaging of Rep reveals the dynamic interplay between an accessory replicative helicase and the replisome. Nucleic Acids Res. **47**, 6287-6298. (10.1093/nar/gkz298) 31028385 PMC6614839

[44] Jin X et al. 2021 Membraneless organelles formed by liquid-liquid phase separation increase bacterial fitness. Sci. Adv. **7**, eabh2929. (10.1126/sciadv.abh2929) 34669478 PMC8528417

[45] Miller H, Cosgrove J, Wollman AJM, Taylor E, Zhou Z, ƠToole PJ, Coles MC, Leake MC. 2018 High-speed single-molecule tracking of CXCL13 in the B-follicle. Front. Immunol. **9**, 1073. (10.3389/fimmu.2018.01073) 29872430 PMC5972203

[46] Llorente-Garcia I et al. 2014 Single-molecule *in vivo* imaging of bacterial respiratory complexes indicates delocalized oxidative phosphorylation. Biochim. Biophys. Acta **1837**, 811-824. (10.1016/j.bbabio.2014.01.020) 24513194

[47] Needham SR, Hirsch M, Rolfe DJ, Clarke DT, Zanetti-Domingues LC, Wareham R, Martin-Fernandez ML. 2013 Measuring EGFR separations on cells with ∼10 nm resolution via fluorophore localization imaging with photobleaching. PLoS ONE **8**, e62331. (10.1371/journal.pone.0062331) 23650512 PMC3641073

[48] Lemmon MA. 2009 Ligand-induced ErbB receptor dimerization. Exp. Cell Res. **315**, 638-648. (10.1016/j.yexcr.2008.10.024) 19038249 PMC2667204

[49] Cho HS, Mason K, Ramyar KX, Stanley AM, Gabelli SB, Denney DW, Leahy DJ. 2003 Structure of the extracellular region of HER2 alone and in complex with the Herceptin Fab. Nature **421**, 756-760. (10.1038/nature01392) 12610629

[50] Maadi H, Nami B, Tong J, Li G, Wang Z. 2018 The effects of trastuzumab on HER2-mediated cell signaling in CHO cells expressing human HER2. BMC Cancer **18**, 238. (10.1186/s12885-018-4143-x) 29490608 PMC5831215

[51] Wehrman TS, Raab WJ, Casipit CL, Doyonnas R, Pomerantz JH, Blau HM. 2006 A system for quantifying dynamic protein interactions defines a role for Herceptin in modulating ErbB2 interactions. Proc. Natl Acad. Sci. USA **103**, 19 063-19 068. (10.1073/pnas.0605218103) PMC1748177 17148612

[52] Saffman PG, Delbrück M. 1975 Brownian motion in biological membranes. Proc. Natl Acad. Sci. USA **72**, 3111-3113. (10.1073/pnas.72.8.3111) 1059096 PMC432930

[53] Claus J et al. 2018 Inhibitor-induced HER2-HER3 heterodimerisation promotes proliferation through a novel dimer interface. Elife **7**, e32271. (10.7554/eLife.32271) 29712619 PMC5929906

[54] Rockberg J, Schwenk JM, Uhlén M. 2009 Discovery of epitopes for targeting the human epidermal growth factor receptor 2 (HER2) with antibodies. Mol. Oncol. **3**, 238-247. (10.1016/j.molonc.2009.01.003) 19393584 PMC5527853

[55] Hulme EC, Trevethick MA. 2010 Ligand binding assays at equilibrium: validation and interpretation. Br. J. Pharmacol. **161**, 1219-1237. (10.1111/j.1476-5381.2009.00604.x) 20132208 PMC3000649

[56] Sorkin A, Duex JE. 2010 Quantitative analysis of endocytosis and turnover of epidermal growth factor (EGF) and EGF receptor. Curr. Protoc. Cell Biol. **46**, 15.14.1-15.14.20. (10.1002/0471143030.cb1514s46) PMC2878126 20235100

[57] Lemmon MA, Bu Z, Ladbury JE, Zhou M, Pinchasi D, Lax I, Engelman DM, Schlessinger J. 1997 Two EGF molecules contribute additively to stabilization of the EGFR dimer. EMBO J. **16**, 281-294. (10.1093/emboj/16.2.281) 9029149 PMC1169635

[58] Odaka M, Kohda D, Lax I, Schlessinger J, Inagaki F. 1997 Ligand-binding enhances the affinity of dimerization of the extracellular domain of the epidermal growth factor receptor. J. Biochem. **122**, 116-121. (10.1093/oxfordjournals.jbchem.a021718) 9276679

[59] Domagala T et al. 2000 Stoichiometry, kinetic and binding analysis of the interaction between epidermal growth factor (EGF) and the extracellular domain of the EGF receptor. Growth Factors **18**, 11-29. (10.3109/08977190009003231) 10831070

[60] Ogiso H et al. 2002 Crystal structure of the complex of human epidermal growth factor and receptor extracellular domains. Cell **110**, 775-787. (10.1016/S0092-8674(02)00963-7) 12297050

[61] Garrett TPJ et al. 2002 Crystal structure of a truncated epidermal growth factor receptor extracellular domain bound to transforming growth factor alpha. Cell **110**, 763-773. (10.1016/S0092-8674(02)00940-6) 12297049

[62] Cho HS, Leahy DJ. 2002 Structure of the extracellular region of HER3 reveals an interdomain tether. Science **297**, 1330-1333. (10.1126/science.1074611) 12154198

[63] Arkhipov A et al. 2013 Architecture and membrane interactions of the EGF receptor. Cell **152**, 557-569. (10.1016/j.cell.2012.12.030) 23374350 PMC3680629

[64] Iino R, Koyama I, Kusumi A. 2001 Single molecule imaging of green fluorescent proteins in living cells: E-cadherin forms oligomers on the free cell surface. Biophys. J. **80**, 2667-2677. (10.1016/S0006-3495(01)76236-4) 11371443 PMC1301454

[65] Zhao W, Pan Y, Wu J, Cai M, Tian Y, Xu H, Yu L, Wang H. 2014 Mapping the resting and stimulated EGFR in cell membranes with topography and recognition imaging. Anal. Methods **6**, 7689-7694. (10.1039/C4AY00995A)

[66] Zanetti-Domingues LC et al. 2018 The architecture of EGFR's basal complexes reveals autoinhibition mechanisms in dimers and oligomers. Nat. Commun. **9**, 4325. (10.1038/s41467-018-06632-0) 30337523 PMC6193980

[67] Zhang F, Wang S, Yin L, Yang Y, Guan Y, Wang W, Xu H, Tao N. 2015 Quantification of epidermal growth factor receptor expression level and binding kinetics on cell surfaces by surface plasmon resonance imaging. Anal. Chem. **87**, 9960-9965. (10.1021/acs.analchem.5b02572) 26368334 PMC4836855

[68] Wang Z, Zhang L, Yeung TK, Chen X. 1999 Endocytosis deficiency of epidermal growth factor (EGF) receptor–ErbB2 heterodimers in response to EGF stimulation. Mol. Biol. Cell **10**, 1621-1636. (10.1091/mbc.10.5.1621) 10233167 PMC30486

[69] Nussinov R, Tsai CJ, Jang H. 2019 Oncogenic KRas mobility in the membrane and signaling response. Semin. Cancer Biol. **54**, 109-113. (10.1016/j.semcancer.2018.02.009) 29499269

[70] Stabley D, Retterer S, Marshall S, Salaita K. 2013 Manipulating the lateral diffusion of surface-anchored EGF demonstrates that receptor clustering modulates phosphorylation levels. Integr. Biol. **5**, 659-668. (10.1039/c3ib20239a) PMC3609930 23416883

[71] Sousa LP, Lax I, Shen H, Ferguson SM, Camilli PD, Schlessinger J. 2012 Suppression of EGFR endocytosis by dynamin depletion reveals that EGFR signaling occurs primarily at the plasma membrane. Proc. Natl Acad. Sci. USA **109**, 4419-4424. (10.1073/pnas.1200164109) 22371560 PMC3311323

[72] Paez JG et al. 2004 EGFR mutations in lung, cancer: correlation with clinical response to gefitinib therapy. Science **304**, 1497-1500. (10.1126/science.1099314) 15118125

[73] Lajoie MJ et al. 2020 Designed protein logic to target cells with precise combinations of surface antigens. Science **369**, 1637-1643. (10.1126/science.aba6527) 32820060 PMC8085813

[74] Aoyagi K, Kouhuji K, Kizaki J, Isobe T, Hashimoto K, Shirouzu K. 2014 Molecular targeting to treat gastric cancer. World J. Gastroenterol. **20**, 13 741-13 755. (10.3748/wjg.v20.i38.13741) PMC4194558 25320512

[75] Wollman AJM et al. 2022 Critical roles for EGFR and EGFR–HER2 clusters in EGF binding of SW620 human carcinoma cells. *FigShare*. (10.6084/m9.figshare.c.5965943) PMC9131850 35612280

